# Predictive Path-Tracking Control of an Autonomous Electric Vehicle with Various Multi-Actuation Topologies

**DOI:** 10.3390/s24051566

**Published:** 2024-02-28

**Authors:** Chenhui Lin, Boyuan Li, Efstathios Siampis, Stefano Longo, Efstathios Velenis

**Affiliations:** 1 Advanced Vehicle Engineering Centre, Cranfield University, Bedford MK43 0AL, UK; liboyuan@zhejianglab.com (B.L.); efstathios.siampis@cranfield.ac.uk (E.S.); s.longo@cranfield.ac.uk (S.L.); e.velenis@cranfield.ac.uk (E.V.); 2 Research Center for Intelligent Transportation, Zhejiang Lab, Hangzhou 311121, China

**Keywords:** autonomous vehicle, path tracking, multi-actuation, predictive control

## Abstract

This paper presents the development of path-tracking control strategies for an over-actuated autonomous electric vehicle. The vehicle platform is equipped with four-wheel steering (4WS) as well as torque vectoring (TV) capabilities, which enable the control of vehicle dynamics to be enhanced. A nonlinear model predictive controller is proposed taking into account the nonlinearities in vehicle dynamics at the limits of handling as well as the crucial actuator constraints. Controllers with different actuation formulations are presented and compared to study the path-tracking performance of the vehicle with different levels of actuation. The controllers are implemented in a high-fidelity simulation environment considering scenarios of vehicle handling limits. According to the simulation results, the vehicle achieves the best overall path-tracking performance with combined 4WS and TV, which illustrates that the over-actuation topology can enhance the path-tracking performance during conditions under the limits of handling. In addition, the performance of the over-actuation controller is further assessed with different sampling times as well as prediction horizons in order to investigate the effect of such parameters on the control performance, and its capability for real-time execution. In the end, the over-actuation control strategy is implemented on a target machine for real-time validation. The control formulation proposed in this paper is proven to be compatible with different levels of actuation, and it is also demonstrated in this work that it is possible to include the particular over-actuation formulation and specific nonlinear vehicle dynamics in real-time operation, with the sampling time and prediction time providing a compromise between path-tracking performance and computational time.

## 1. Introduction

Over the past few decades, due to the increasing demands to improve the safety, efficiency and comfort of road vehicles, autonomous vehicles (AVs) have been widely considered as the next generation of road transportation. The control techniques for AVs have been rapidly developed with a great deal of research work carried out for various objectives, including path and motion planning [1,2,3], path tracking [4,5], obstacle detection and avoidance [6,7] and so forth. Among these topics, the fundamental function of path tracking, and the real-time realization of it, is the main focus of this paper.

Regarding the path-tracking problem, geometry-based control methods have been raised for active front-wheel steering (FWS) vehicles [8,9]. Geometric path-tracking controllers are able to track a path only with the geometry of vehicle kinematics and of the reference path, but they are less suitable for control at the limits of handling due to the lack of knowledge on vehicle dynamics. In terms of the path-tracking controllers involving vehicle dynamics, Roselli et al. proposed a path-tracking controller for lane keeping based on the H-infinity technique [10]. According to experimental tests, the H-infinity controller was able to achieve an overall smaller lateral error in comparison with a PID controller with feedforward, leading to a smoother action in the corner. In another study, a higher-order sliding mode controller was designed for lateral control, and robustness was validated by the test results [11]. In addition to these, optimal control theory has been introduced to the scope. Lee et al. proposed an optimal path-tracking controller based on linear quadratic Gaussian control, which provided better performance than the geometry-based controllers in terms of tracking deviation [12].

Among various control methods, model predictive control (MPC) has been found to be an outstanding technique for autonomous vehicle control. In [13], various controllers including a geometric controller, linear quadratic regulator and MPC were compared in terms of their path-tracking performance, and the simulation results demonstrated that the best path-tracking performance was achieved by MPC with the minimum control effort. Yakub et al. [14] extended the study by comparing the tracking performance of MPC and linear quadratic control for different speeds, tyre–road friction coefficients and control topologies, including FWS, four-wheel steering (4WS) and FWS with direct yaw moment control (DYC). A similar conclusion was drawn that MPC is more suitable for multi-variable systems. An additional advantage of MPC is that it takes into consideration physical constraints present in the system, such as state ranges, input limitation and road boundaries [3,15]. Therefore, MPC has been extensively applied in research work for the path-tracking control of autonomous vehicles [16,17,18,19,20]. In [16], Raffo et al. presented an MPC controller for path tracking. The controller was based on a linear model, and a constant velocity was assumed in order to neglect the vehicle’s longitudinal dynamics. Another path-tracking controller was developed in [21] with an adaptive preview strategy, where it was demonstrated by the simulation results that the tracking error significantly increased when the vehicle approached the limits of handling condition. In the above studies, linear vehicle dynamics models are applied for control development. This could reduce the complexity of the MPC optimization problem, while on the other hand, when it comes to situations where the vehicle is equipped with multi-actuation, or is operating at extreme conditions, a linear model may no longer provide a good prediction of the vehicle dynamics as the vehicle behaviour becomes highly nonlinear.

In addition to active FWS control, techniques like DYC, torque vectoring (TV) in particular, have been extensively discussed in the literature for vehicle control. Typically, TV refers to the differential technique that varies the torque delivered on each wheel. With the development of electric vehicles (EVs), the application of independent in-wheel motors provides a more straightforward realization of TV. Various path-tracking controllers were compared in [22], some of which applied TV, while the others did not have the functionality. The results showed that the cornering response of the vehicle could be effectively improved with the application of TV, especially at the limits of handling condition, and this was due to the generation of a direct yaw moment helping in the stabilization of the vehicle. Hence, TV has been applied in several studies for the development of driver assist systems, aiming to improve the vehicle performance and guarantee a consistently safe and stable cornering response [23,24,25]. There is no doubt that AV control can benefit from the multi-actuation formulation, referring to the integration of TV and steering. Chen et al. developed a path-tracking controller for vehicles with four-wheel drive (4WD) as well as 4WS [26]. With a hierarchical structure, the control demand for path-tracking as well as lateral stabilization was obtained in the higher level controller, and tyre force allocation was then carried out at a lower level to achieve that. In [27], another path-tracking controller was proposed based on MPC. The controller was also hierarchical, based on an LTV system, and could be implemented in real time. The disadvantage of such a formulation is that the results may not be optimal as the control inputs are not directly integrated into the high-level strategies. What is more, as the higher levels were based on a linear system, it was hard to guarantee feasibility, especially in extreme conditions. This could hinder the vehicle from operating at the limits of handling. In [28], Acosta et al. developed a multi-actuation controller for autonomous drift control. The controller was based on nonlinear vehicle and tyre models, and the results showed that the dynamical capability of a vehicle could be exploited by combining FWS and TV. However, a PID controller was used instead of MPC for the path-tracking purpose, which meant that the vehicle might not be able to track a complicated path properly. In addition, the controller might not be able to be implemented in real time. To the best of our knowledge, there have been few studies on multi-actuation control including rear-wheel steering (RWS), which is part of the novelty of our work.

In this paper, we present the development of path-tracking control strategies for an over-actuated autonomous EV, which is equipped with 4WS as well as TV functionality on the rear axle. The vehicle is supposed to track a desired path at a specified reference velocity, which is close to the maximum possible velocity according to the turning radius and road–tyre friction coefficient. The control design is based on nonlinear MPC (NMPC) so that the nonlinearities of the vehicle dynamics are taken into account, which is beneficial to the operation at the limits of handling. Four controllers are built and tested in this work, including FWS only, FWS with TV (FWS-TV), 4WS and 4WS with TV (4WS-TV). The four controllers are compared in terms of path-tracking performance to validate the advantage of the over-actuation topology. Further study has also been carried out to implement the proposed over-actuation controller in real time. The control performance of the 4WS-TV controller with different prediction horizons as well as sampling time is investigated and discussed. For the purpose of the real-time implementation of the controller, a compromise has been made between path-tracking performance and computational time, and an appropriate setup of prediction horizon and sampling time is identified. In the end, the control strategy is implemented on a real-time target machine for validation. The major contribution of this paper can be summarized in two parts. First, this paper proposes a control formulation based on NMPC that is compatible with different levels of actuation, and the control performance with those actuation topologies has been compared and studied. The results demonstrate that the over-actuation formulation with both TV and 4WS leads to the best path-tracking performance for an autonomous vehicle at the limits of handling, in comparison with the individual utilization of either TV or 4WS. The second contribution of this paper is the demonstration that the proposed controller including the particular over-actuation formulation as well as nonlinear vehicle dynamics can be implemented in real time. An appropriate prediction horizon and sampling time have been discovered for the controller, which strikes a balance between path-tracking performance and system complexity. Furthermore, the controller is implemented and validated in a real-time target machine to prove that it can be executed in real time. It is worth mentioning that although the work presented in this paper is based on simulation only, some practical testing has been carried out to validate the proposed 4WS-TV control strategy. Both simulation and experimental results for tracking the same reference path are demonstrated and compared in [29], which confirms the effectiveness of the controller as well as its capability of real-time implementation.

This paper is organized as follows. Section 2 introduces the modelling of vehicle dynamics. Then the optimal control problem is formulated in Section 3. Section 4 presents the simulation results of the controllers. In Section 4.1, the path-tracking performances of the four controllers are demonstrated and compared. In Section 4.2, the path-tracking performance of the over-actuated controller with different sampling times and prediction horizons is demonstrated. Finally, we demonstrate the implementation of the controller in a real-time target machine.

## 2. Vehicle Dynamics Modelling

**Figure 1:**
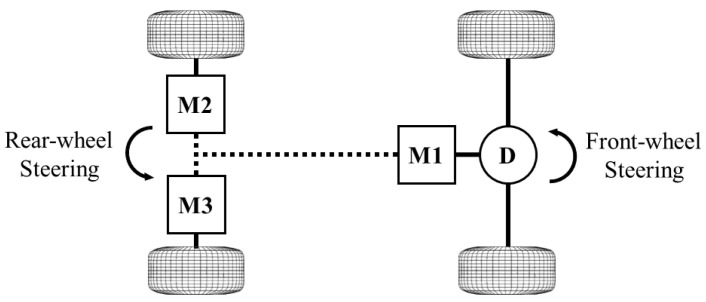
Actuator topology of the case study vehicle.

Figure 1 shows the actuator topology of the specific vehicle for which the controller in this paper is designed. The vehicle in this study is a 4WD EV prototype developed by Delta Cosworth for the AID-CAV project. The powertrain includes three electric motors, responsible for both the acceleration and deceleration of the vehicle. The motor M1 drives the front wheels through an open differential on the front axle, and the rear wheels are, respectively, driven by the other two motors M2 and M3, with which torque vectoring is performed. In addition, the autonomous EV is equipped with 4WS functionality, realized by two steer-by-wire systems. It should be mentioned that the control strategies we have developed for this specific vehicle are extendable to fit most kinds of multi-actuation configuration of EVs, like an individual hub motor for each wheel.

### 2.1. Equations of Motion

**Figure 2:**
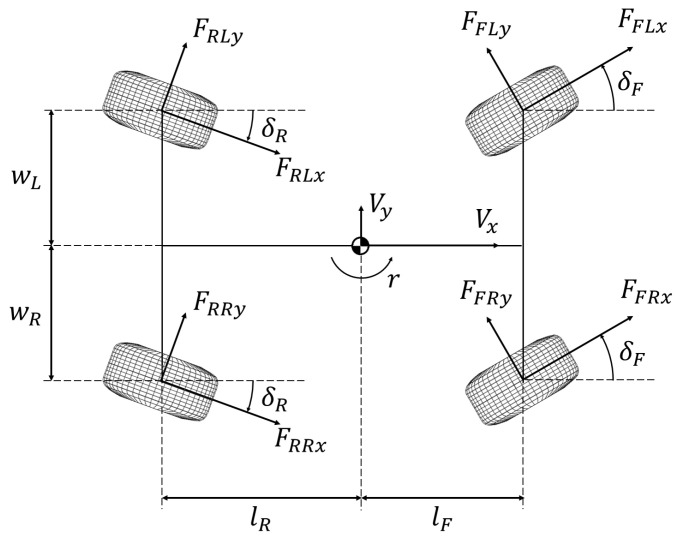
Schematic diagram of the vehicle model.

A two-track vehicle model is applied for the design of the path-tracking controller. The model is formulated around the vehicle’s centre of gravity (CoG), and with assumptions that the vehicle travels on a horizontal plane, while the pitch, roll and heave motion are neglected. The model is shown in Figure 2. Focusing on the longitudinal, lateral and yaw motion of the vehicle, the equations of motion can be derived based on Newton–Euler equations using the present tyre forces, and are given as follows:

(1) $$\begin{array}{cc} m(\dot{V_{x}}-V_{y}r)\;=\; & \left(F_{FLx}+F_{FRx}\right)\cos\delta_{F}-\left(F_{FLy}+F_{FRy}\right)\sin\delta_{F}\\ + & \left(F_{RLx}+F_{RRx}\right)\cos\delta_{R}-\left(F_{RLy}+F_{RRy}\right)\sin\delta_{R} \end{array}$$

(2) $$\begin{array}{cc} m(\dot{V_{y}}+V_{x}r)\;=\; & \left(F_{FLx}+F_{FRx}\right)\sin\delta_{F}-\left(F_{FLy}+F_{FRy}\right)\cos\delta_{F}\\ + & \left(F_{RLx}+F_{RRx}\right)\sin\delta_{R}-\left(F_{RLy}+F_{RRy}\right)\cos\delta_{R} \end{array}$$

(3) $$\begin{array}{cc} I_{z}\dot{r}\;=\; & l_{F}\cdot \left(F_{FLx}+F_{FRx}\right)\sin\delta_{F}+l_{F}\cdot \left(F_{FLy}+F_{FRy}\right)\cos\delta_{F}\\ - & l_{R}\cdot \left(F_{RLx}+F_{RRx}\right)\sin\delta_{R}-l_{R}\cdot \left(F_{RLy}+F_{RRy}\right)\cos\delta_{R}\\ - & w_{L}\cdot \left(F_{FLx}\cos\delta_{F}-F_{FLy}\sin\delta_{F}\right)\\ - & w_{L}\cdot \left(F_{RLx}\cos\delta_{R}-F_{FLy}\sin\delta_{R}\right)\\ + & w_{R}\cdot \left(F_{FRx}\cos\delta_{F}-F_{FRy}\sin\delta_{F}\right)\\ + & w_{R}\cdot \left(F_{RRx}\cos\delta_{R}-F_{RRy}\sin\delta_{R}\right), \end{array}$$

where *m* and $I_{z}$ stand for the mass of the vehicle and its moment of inertia about the vertical axis through the CoG. $l_{F}$ and $l_{R}$ represent the distances from the CoG to the front and rear axle, while $w_{L}$ and $w_{R}$ refer to the left and right portions of the track width divided by the CoG. The longitudinal velocity, lateral velocity and yaw rate of the vehicle are denoted by $V_{x}$, $V_{y}$ and *r*, respectively. $F_{ijk}$(i=F,R,j=L,R,k=x,y) stand for the longitudinal and lateral tyre forces, while $\delta_{F}$ and $\delta_{R}$ represent the steering angles of the vehicle on the front and rear wheels, respectively.

For the path-tracking purpose, it is fundamental to include the vehicle’s position in the model. In this paper, the vehicle’s position is identified in the Cartesian coordinate system, and the derivatives of the vehicle’s position coordinates *X* and *Y* as well as yaw angle Ψ can be calculated as:

(4) $$\dot{X}=V_{x}\cos\left(\Psi \right)-V_{y}\sin\left(\Psi \right)$$

(5) $$\dot{Y}=V_{x}\sin\left(\Psi \right)+V_{y}\cos\left(\Psi \right)$$

(6) $$\dot{\Psi }=r$$

### 2.2. Tyre Model

When operating near the adhesive limits, vehicle dynamics become highly nonlinear due to the characteristics of tyre force. Thus, it is important to apply an appropriate tyre model when developing control strategies for such extreme operating conditions. Since control development is focused on planar motion, the pitch and roll of the vehicle as well as the vertical motion of the sprung mass can be neglected. Under these assumptions, the vertical load at each wheel can be calculated as a combination of the static weight on each corner and the transferred load resulting from both longitudinal and lateral acceleration. The total vertical loads at each wheel $F_{ijz}$ are given by:

(7) $$F_{FLz}=F_{FLz0}+\frac{mh}{lw}\cdot \left(-w_{R}a_{x}-l_{R}a_{y}\right)$$

(8) $$F_{FRz}=F_{FRz0}+\frac{mh}{lw}\cdot \left(-w_{L}a_{x}+l_{R}a_{y}\right)$$

(9) $$F_{RLz}=F_{RLz0}+\frac{mh}{lw}\cdot \left(w_{R}a_{x}-l_{F}a_{y}\right)$$

(10) $$F_{RRz}=F_{RRz0}+\frac{mh}{lw}\cdot \left(w_{L}a_{x}-l_{F}a_{y}\right),$$

where $a_{x}$ and $a_{y}$ are the longitudinal and lateral acceleration of the vehicle, and *h* denotes the height of the CoG from the ground. $F_{ijz0}$ are the static vertical forces on each wheel.

The side slip angles on the front and rear tyres can be calculated by the following equations. It is assumed that the side slip angles are the same at the left and right tyres.

(11) $$\alpha_{F}=\arctan\frac{V_{y}+l_{F}\cdot r}{V_{x}}-\delta_{F}$$

(12) $$\alpha_{R}=\arctan\frac{V_{y}-l_{R}\cdot r}{V_{x}}-\delta_{R}$$

By applying appropriate constraints on the control inputs, an assumption can be made that the tyres do not go beyond their adhesion limit in the longitudinal direction, and hence, the rotational dynamics of the wheels can be neglected [30]. In this case, the longitudinal tyre force is supposed to be proportional to the driving or braking torque applied on the wheels. As mentioned in Section 1, the front wheels are driven by a single motor through an open differential; the torque input on the front axle is evenly distributed on the two front wheels. With $T_{F}$, $T_{RL}$ and $T_{RR}$ representing the three torque inputs from motors M1, M2 and M3, the longitudinal tyre force of the vehicle on each wheel can be calculated as follows:

(13) $$F_{Fjx}=\frac{T_{F}/2}{R_{w}}$$

(14) $$F_{Rjx}=\frac{T_{Rj}}{R_{w}}$$

The maximum tyre force can be calculated as

(15) $$F_{ij,max}=\mu \cdot F_{ijz},$$

where μ refers to the tyre–road friction coefficient. To maintain the crucial coupling between longitudinal and lateral tyre forces, the maximum available lateral tyre force is determined by the friction circle

(16) $$F_{ijy,max}=\sqrt{{F_{ij,max}}^{2}-{F_{ijz}}^{2}}$$

Thus, the lateral tyre force on individual wheels can be calculated by the simplified Pacejka’s Magic Formula tyre model [31]

(17) $$F_{ijy}=-F_{ijz}\cdot D\sin\left(C\arctan\left(B\alpha_{i}\right)\right)$$

## 3. Predictive Path-Tracking Controller

The nonlinear continuous-time system can be described as:

(18) $${\dot{x}}_{t}=fc\left(x_{t},\;u_{t}\right),$$

where $x_{t}$ stands for the state vector ${\left[V_{x},V_{y},r,X,Y,\Psi \right]}^{T}$ and *u* refers to the control input vector. With regards to the actuation configuration, there are four formulations to be investigated in this paper, including FWS, FWS-TV, 4WS and 4WS-TV. For the FWS and FWS-TV formulations, the RWS angle is not included in the controller and thus remains zero. For the FWS and 4WS formulations, it is assumed that the driving torque delivered on each wheel equals the same, which means the torque from the three motors have the relationship:

(19) $$\frac{T_{F}}{2}=T_{RL}=T_{RR}=T_{w},$$

where $T_{w}$ is the actual control input in the FWS and 4WS formulations. Table 1 shows the actuation configuration of each formulation, and the control input vector *u* of each of them is shown as follows:

(20) $$\begin{array}{cc} u_{FWS} & ={\left[\delta_{F},T_{w}\right]}^{T}\\ u_{FWS-TV} & ={\left[\delta_{F},T_{F},T_{RL},T_{RR}\right]}^{T}\\ u_{4WS} & ={\left[\delta_{F},\delta_{R},T_{w}\right]}^{T}\\ u_{4WS-TV} & ={\left[\delta_{F},\delta_{R},T_{F},T_{RL},T_{RR}\right]}^{T} \end{array}$$

The controller development is based on NMPC, and is realized in the sampled-data framework by discretizing the nonlinear continuous-time system with the explicit Runge–Kutta 4th order method. The main purpose of the controller is to follow the reference path at the reference velocity, and the discrete NMPC problem is formulated as

(21) $$\begin{array}{cc} \min_{x,u} & \sum_{k=0}^{N-1}{\left(x_{k+1}-x_{ref,k+1}\right)}^{T}Q\left(x_{k+1}-x_{ref,k+1}\right)+{u_{k}}^{T}Ru_{k}\\ st.\;\; & x_{0}=x_{initial}\\ \; & x_{k+1}=f_{d}\left(x_{k},u_{k}\right),k=0,\cdots ,N-1\\ \; & x_{min}\leq x_{k}\leq x_{max},k=0,\cdots ,N-1\\ \; & u_{min}\leq u_{k}\leq u_{max},k=0,\cdots ,N-1, \end{array}$$

where *N* is the prediction horizon steps, $x_{ref}$ is the reference for state vector *x*, namely ${\left[V_{x,ref},V_{y,ref},r_{ref},X_{ref},Y_{ref},\Psi_{ref}\right]}^{T}$, and *Q*, *R* are the weighting matrices of the state and control input vectors, respectively. $f_{d}$ represents the discrete-time system obtained from $f_{c}$. In terms of the constraints on control commands, it is worth mentioning that despite the use of box constraints in this paper, the formulation can easily take a time-varying constraint on the control inputs.

The reference path is parametrized by the arc length *S* along the path from the origin point, where S∈[0,L] and *L* is the total length of the path. With this parameterization, the position $X_{ref}\left(S\right)$, $Y_{ref}\left(S\right)$ of any point on the reference path can be obtained by calculating the third order polynomial for the argument S. In addition, the tangential angle of the path at the point can be obtained as

(22) $$\Psi_{ref}\left(S\right)=\arctan\frac{\partial Y_{ref}\left(S\right)}{\partial X_{ref}\left(S\right)},$$

and is used as the reference yaw angle of the vehicle. This parameterization takes advantage of the known waypoints on the reference path and provides an accurate enough interpolation within them [20].

For path-tracking purposes, the relative position of the vehicle with regards to the reference path is required. Point $\left(X_{ref}\left(S_{0}\right),Y_{ref}\left(S_{0}\right)\right)$ is proposed as the projection of the vehicle position on the reference path, and $S_{0}$ can be obtained by solving the optimization problem

(23) $$S_{0}=\min_{S}\;\sqrt{{\left[X-X_{ref}\left(S\right)\right]}^{2}+{\left[Y-Y_{ref}\left(S\right)\right]}^{2}}.$$

$S_{0}$ can be used to denote the progress of the vehicle along the reference path, and the distance between the vehicle and this projection point refers to the lateral deviation of the vehicle from the path. In order to reduce the computational time, the solving of this optimization problem is carried out in a local range, which means only the waypoints within a specific range are included in the problem. The range is identified based on the vehicle’s previous position and is proportional to the velocity. This could massively increase the efficiency of localization while maintaining an accurate solution under the assumption that the vehicle does not deviate far from the reference path.

**Figure 3:**
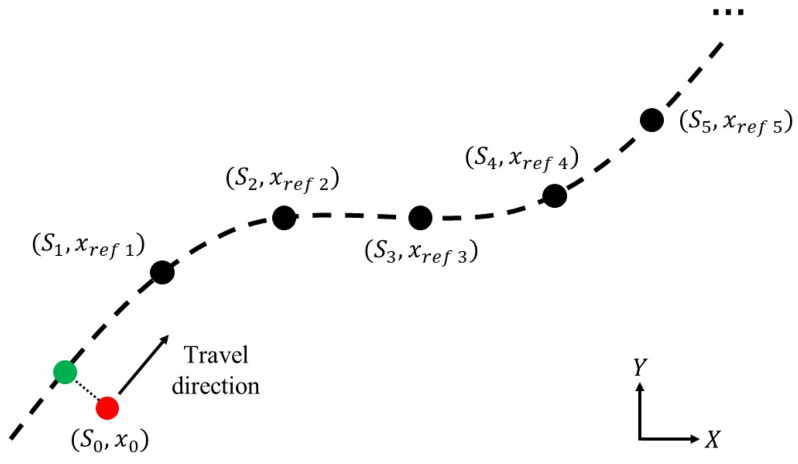
Diagram of the reference waypoints. The red point represents the vehicle’s position, while the green point stands for the projection of the vehicle position on the reference path. The interval of the waypoints $\Delta S=V_{ref}\cdot t_{s}$, where $V_{ref}$ is the reference velocity and $t_{s}$ is the sampling time of the controller.

For the discrete objective function, a total of *N* waypoints are required to generate $x_{ref}$. The waypoints are supposed to follow the projection point $\left(X_{ref}\left(S_{0}\right),Y_{ref}\left(S_{0}\right)\right)$, with an interval of ΔS,

(24) $$\Delta S=S_{k+1}-S_{k}=V_{ref}\cdot t_{s},k=0,\cdots ,N-1,$$

where $V_{ref}$ is the reference velocity and $t_{s}$ is the sampling time of the discrete-time system. Figure 3 shows a diagram of the generated waypoints based on the feedback states of the vehicle. The reference state vector $x_{ref}$ is then evaluated by carrying out third-order spline polynomials based on the argument *S*.

**Figure 4:**
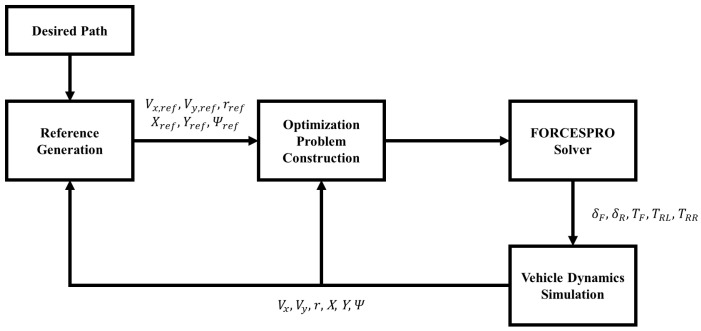
Diagram of the control system.

Figure 4 shows the diagram of the complete control algorithm. The parameterization of the desired path is completed offline prior to the simulation. The solvers for the optimization problems are generated with FORCESPRO [32,33], which is an MPC and embedded optimization solution developed by Embotech.

## 4. Simulation Results

In this section, we demonstrate the path-tracking performance of the vehicle with different control formulations and configurations according to the simulation results. Except for the real-time implementation, the simulation is carried out in the MATLAB Simulink environment. The generated NMPC solvers are imported into the Simulink model, which is connected to IPG CarMaker to provide a vehicle dynamics simulation with a high-fidelity vehicle model and scenario. All the simulation sessions are run on a workstation laptop (Intel Core i7-8750H CPU at 2.2 GHz with 32 GB RAM).

**Figure 5:**
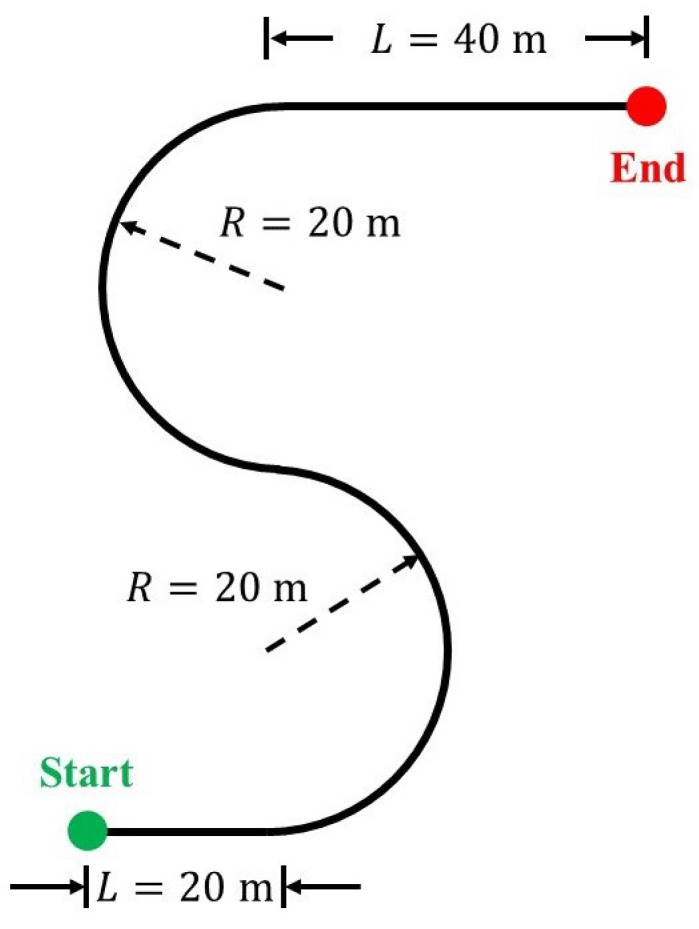
Reference path for the vehicle to follow. Here, *L* refers to the straight length, and *R* refers to the turning radius.

In this paper, a double U-turn scenario as shown in Figure 5 is used for testing the control performance. The desired path consists of two connected U-turns, with two straights before and after them, and the radius of both the U-turns is 20 m. Instead of the double lane change manoeuvre that is commonly used to examine vehicle stability, the double U-turn scenario is adopted in this work as the fixed curvature helps to better capture the difference in path-tracking performance brought by the different levels of actuation. In addition, the step change in curvature in the middle of the double U-turns requires the vehicle to turn sharply for path-tracking, which is challenging in terms of vehicle response and stability, especially when the vehicle is operating close to the limits of handling. This helps us to understand and compare the vehicle’s flexibility with different controllers by seeing how close the vehicle can get to this path. In the simulation, the reference velocity of the vehicle is 52 kph, which is close to the handling limits of the vehicle corresponding to the turning radius as well as the maximum tyre–road friction coefficient. This can well demonstrate the control performance at the limits of handling, and can thus better visualize the difference among all actuation topologies. Table 2 shows the parameters of the above vehicle and tyre models. The weighting factors of MPC for each controller can be found in Equations (25) and (26), and they are obtained by scaling down the relative weight of each variable with their respective scaling factor. The scaling factors equal the square of each variable’s reasonable value and bring the different terms in the objective function to a similar level, while the relative weights are used to reflect the priority of each variable. In this work, more penalization was put on position X and Y deviation, as well as the control inputs. In order to compare the performance of different actuation topologies, it is ensured that each variable’s weighting factor is consistent across all topologies.

(25) $$\begin{array}{cc} Q & =diag([50.0,50.0,16.4,100,100,328.3]) \end{array}$$

(26) $$\begin{array}{cc} R_{FWS} & =diag([9848.4,0.0011])\\ R_{FWS-TV} & =diag([9848.4,0.00031,0.0011,0.0011])\\ R_{4WS} & =diag([9848.4,9848.4,0.0011])\\ R_{4WS-TV} & =diag([9848.4,9848.4,0.00031,0.0011,0.0011]) \end{array}$$

### 4.1. Path-Tracking Performance of Different Actuation Topologies

**Figure 6:**
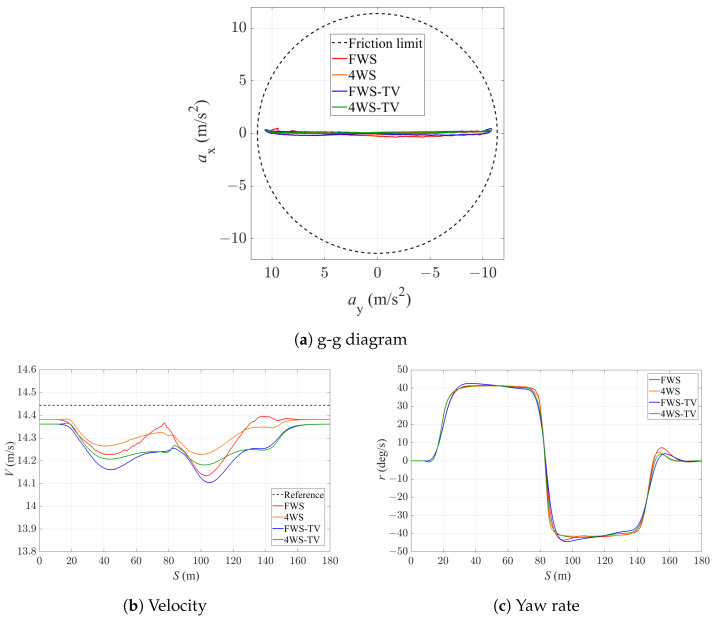
States of the vehicle with the four controllers.

In this part, the simulation results are presented regarding the path-tracking performance of the vehicle with the four controllers, which have a sampling time of 0.02 s and a prediction horizon of 1 s. The acceleration diagram of the vehicle during the path-tracking process is shown in Figure 6a. It can be seen that the vehicle is quite close to the acceleration limit identified by the tyre friction circle, which indicates that the vehicle is at the limits of handling with all the four controllers at the double U-turns. Figure 6b shows the velocity-tracking performance of the vehicle. A larger velocity deviation takes place during the sharp turn, but with the 4WS-TV controller, the vehicle tends to have the smallest velocity variation during the double U-turns. It can also be noticed that there is some steady-state deviation on the straights, which is due to the presence of penalization on torque inputs in the objective function. If the steady-state deviation is to be further reduced, more torque inputs would be required. This would lead to a larger overall objective and would not be the optimal solution from the controller’s perspective. Figure 6c shows the yaw rate of the vehicle with different controllers. It can be noticed that with the FWS and FWS-TV controllers, there is a larger overshoot in the yaw rate in response to the curvature change compared with the 4WS and 4WS-TV controllers. This indicates that the application of 4WS does a better job than TV in terms of reaching the required yaw rate during cornering, while 4WS together with TV can further increase the vehicle’s stability when operating at the limits of handling.

**Figure 7:**
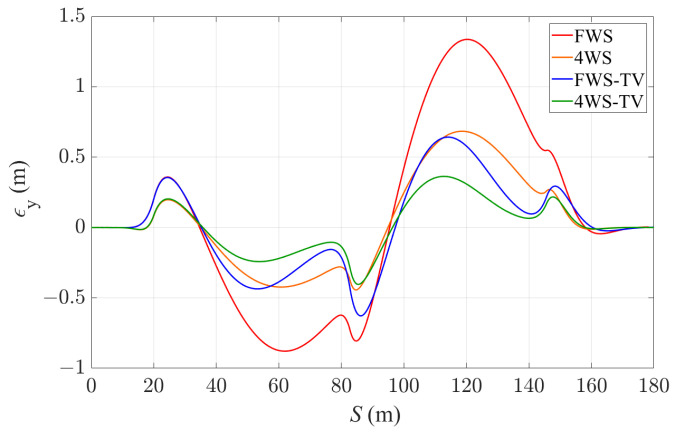
Lateral deviation of the vehicle with the four controllers.

Figure 7 shows the lateral deviation $\varepsilon_{y}$ of the vehicle from the reference path with all the four controllers, and Table 3 provides a summary of the average and maximum lateral deviation with the controllers. In general, the increase in actuation leads to a smaller lateral tracking error. The vehicle obtained the largest lateral deviation up to 1.337 m with the FWS controller in the double U-turn scenario. By comparing the FWS, 4WS and FWS-TV controllers, it can be seen that the application of either RWS or TV can significantly enhance the cornering response and reduce the lateral deviation value, while the utilization of TV seems to provide a better performance than 4WS, with the ability to reduce lateral deviation more quickly. By applying both 4WS and TV, the lateral deviation is reduced most significantly compared to the FWS case. The average tracking error is 73% smaller, and the error is around 0.4 m even at the sharp change in curvature. This proves the advantage of RWS in addition to TV for autonomous vehicle path tracking, particularly when close to the limits of handling. RWS improves the vehicle’s flexibility and potential to deal with emergency scenarios that require sharp turning.

**Figure 8:**
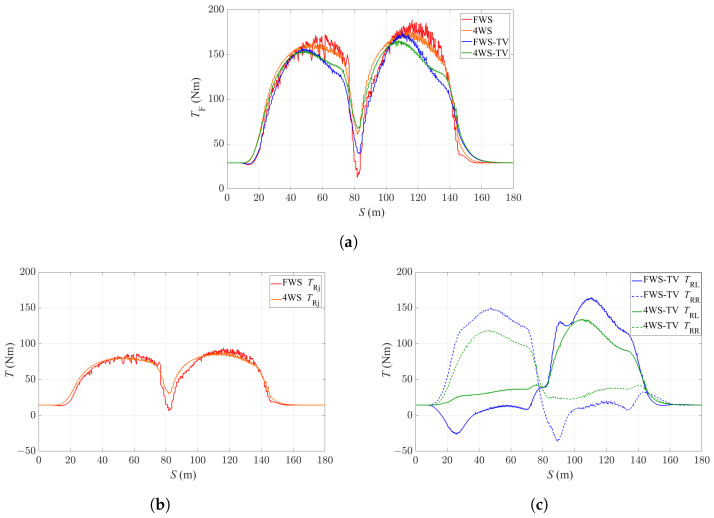
Driving torque commands of the four controllers. (**a**) Front motor torque commands. (**b**) Rear motor torque commands of the FWS and 4WS controllers. (**c**) Rear motor torque commands of the FWS-TV and 4WS-TV controllers.

Figure 8 shows the driving torques of the three motors with the four controllers. It can be observed that there is some oscillation in the driving torque commands, and this is due to the controller making efforts to track the reference velocity at the limits of handling condition. In general, it can seen that there is less oscillation in the torque commands of the FWS-TV and 4WS-TV controllers, which indicates that with the application of TV, it tends to be easier to maintain the vehicle’s handling stability. Furthermore, it can be seen from Figure 8a that the FWS-TV and 4WS-TV controllers require less driving torque on the front axle as a result of TV application. As shown in Figure 8c, the two controllers with TV generate a yaw moment on the rear axle during the turning, which helps to satisfy the required yaw rate, to enhance vehicle stability and thus to improve the path-tracking performance. In addition, due to the application of 4WS, the 4WS-TV controller requires less driving torque on the rear wheels compared with the FWS-TV controller, which requires more torque to compensate for the absence of RWS.

**Figure 9:**
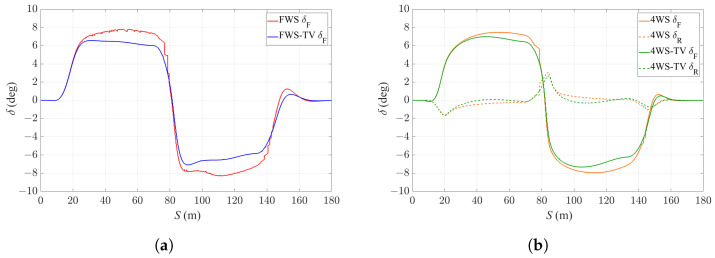
Steering angle commands of the four controllers. (**a**) Steering angle commands of FWS and FWS-TV controllers. (**b**) Steering commands of 4WS and 4WS-TV controllers.

Figure 9 shows the steering angle commands from the four controllers to the vehicle. Figure 9a compares the front steering commands by the FWS and FWS-TV controllers, and it is shown that with the application of TV, the FWS-TV controller requires less front steering angle input than the FWS controller. In Figure 9b, both the 4WS and 4WS-TV controllers tend to have front and rear steering angle in opposite directions in response to the change in curvature, which could generate a larger yaw moment than the FWS and FWS-TV controllers to deal with the harsh change in turning direction. With the utilization of TV, the 4WS-TV controller requires less steering angles on the wheels than the 4WS controller, which is an additional advantage brought by TV.

**Figure 10:**
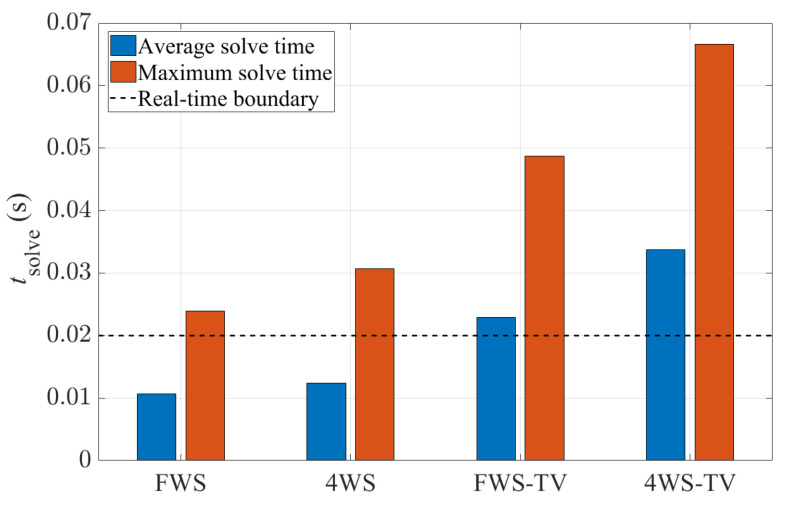
Computational time of the four controllers.

Figure 10 shows the computational time of each controller. Here, the solve time refers to the time that the FORCESPRO solver takes to solve the MPC optimization problem. The FWS controller has an average solve time around 0.01 s and a maximum solve time lower than 0.03 s. In comparison, the 4WS-TV controller has an average solve time longer than 0.03 s and a maximum solve time up to 0.07 s. In general, with an increasing level of actuation, the controller takes a longer computational time due to the system complexity. The average solve times of 4WS, FWS-TV and 4WS-TV are 16%, 115% and 216% higher than that of the FWS controller. The black dashed line in Figure 10 marks the sampling time of the controllers, which is 0.02 s. This determines the maximum solve time allowance for real-time execution, and it can be seen that none of the four proposed controllers can be directly implemented in real time. The FWS and 4WS controllers have the potential for real-time operation if the maximum solve time is capped, while the FWS-TV and 4WS-TV cannot be run in real time with the current parameters.

By comparing the simulation results of the four proposed controllers, it is obvious that both 4WS and TV are able to improve the path-tracking performance of the vehicle. TV improves the vehicle’s response and stability in turning by generating a yaw moment directly. In addition to that, 4WS is able to manipulate the vehicle’s motion with the direction of FWS and RWS angles; thus, the vehicle’s flexibility is increased. The 4WS-TV controller provides the best path-tracking performance among the four formulations, but at the same time, it requires the most computational time among the four controllers and cannot be directly implemented in real time. Hence, further investigation is required on the simplification of the system complexity of the 4WS-TV controller for the purpose of real-time operation.

### 4.2. Path-Tracking Simulation Results with Different Time Parameters

In Section 4.1, the proposed 4WS-TV controller has shown its great advantage for path tracking. On the other hand, the system complexity is preventing the controller from operation in real time. In order to implement the 4WS-TV controller in real time, its system complexity needs to be reduced. One approach to do this while retaining the control formulation is to limit the prediction steps of the MPC formulation. In this part, the control performance of the 4WS-TV controller is further studied with different prediction horizons and sampling times, in order to explore the possibility of the real-time operation of the controller.

**Figure 11:**
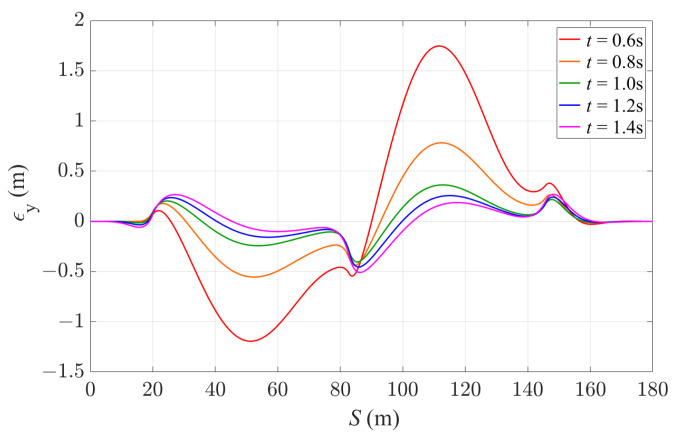
Lateral deviation of the vehicle with 4WS-TV controller with a fixed sampling time of 0.02 s and different prediction horizon times.

First, the effects of prediction horizon on control performance is studied. The sampling time $t_{s}$ is fixed as 0.02 s, and five different prediction horizon times *t* of 0.6 s, 0.8 s, 1.0 s, 1.2 s and 1.4 s are used in the simulation. The lateral deviation of the vehicle is shown in Figure 11 to demonstrate the path-tracking performance, and Table 4 summarizes the average and maximum lateral deviation values with different prediction horizons. As expected, the vehicle achieves a relatively smaller lateral deviation throughout the reference path with a longer prediction horizon, with the average lateral deviation being reduced by 81% with a prediction horizon of 1.4 s, compared to a 0.6 s prediction horizon. This is because a longer prediction horizon allows the controller to plan for a further look-ahead distance, and improves the feasibility to identify the optimal control actions according to the cost function. On the other hand, it can be seen from Table 4 that the maximum lateral deviation value does not change monotonically with the prediction horizon. After the prediction horizon exceeds 1.0 s, the maximum $|\epsilon_{y}|$ value starts increasing with longer prediction horizons. This is because with a longer prediction horizon, the controller carries out control planning in a longer timescale, which potentially leads to a less responsive action in terms of reference tracking.

**Figure 12:**
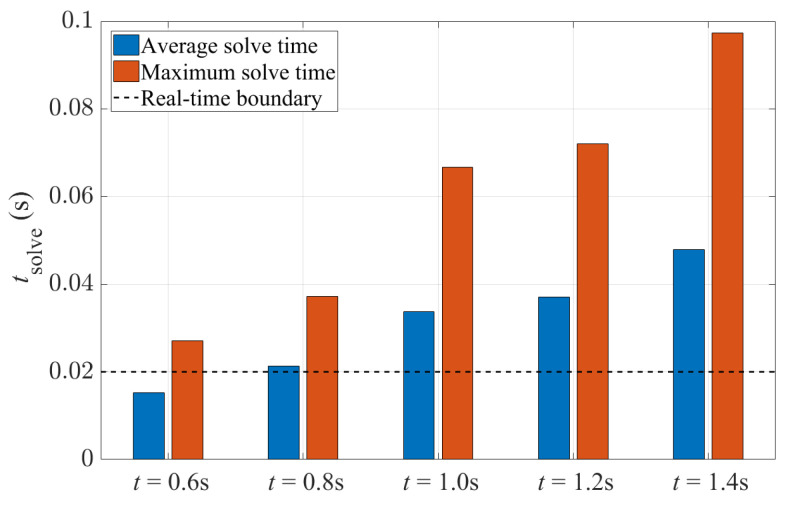
Solve times of the 4WS-TV controller with a fixed sampling time of 0.02 s and different prediction horizon times.

Figure 12 shows the solve time information of the 4WS-TV controller with different prediction horizons. Despite the better path-tracking performance, the controller has a longer solve time with a longer prediction horizon. With a 0.6 s prediction horizon, the controller has the best potential to run in real time, while with a prediction horizon of 1.4 s, the average solve time is 213% higher, and is more than twice of the sampling time. According to Table 4 and Figure 12, it can be inferred that a prediction horizon time of 1.0 s achieves a relatively good balance between control performance and computational time, and thus is probably suitable for the real-time implementation of the 4WS-TV controller. However, the solve time is still higher than the limitation given by the sampling time, so next, the performance is studied with different sampling times.

**Figure 13:**
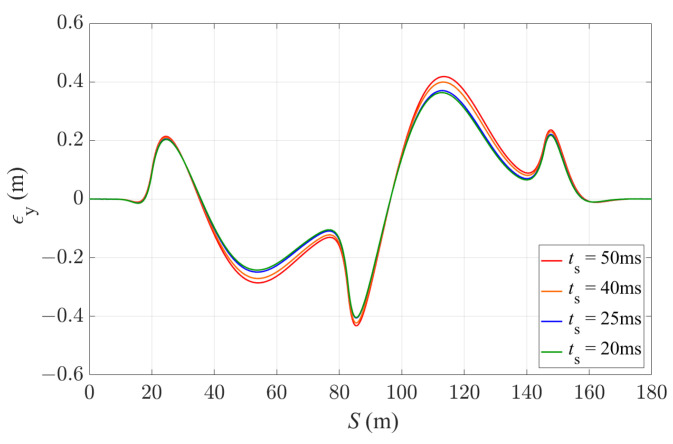
Lateral deviation of the vehicle with 4WS-TV controller with a fixed prediction horizon of 1.0 s and different sampling times.

Next, the prediction horizon time *t* is fixed as 1.0 s, and the sampling time $t_{s}$ is gradually reduced, giving increasing prediction steps of MPC. Four different sampling time are applied here in the simulation, including 0.05 s, 0.04 s, 0.025 s and 0.02 s. As shown in Figure 13 and Table 5, a better prediction of the system dynamics could be achieved with a shorter sampling time; the controller with a 0.02 s sampling time achieves the smallest lateral deviation for path tracking.

**Figure 14:**
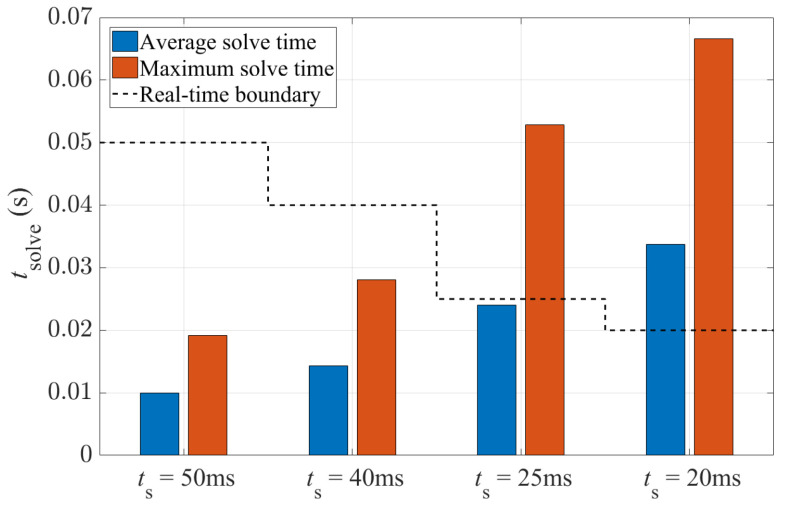
Solve time of the 4WS-TV controller with a fixed prediction horizon of 1.0 s and different sampling times.

However, the difference in path-tracking performance with various sampling times is not significant. The relative difference between the maximum and minimum value of average lateral deviation is 14%, while as shown in Figure 14, there is a large difference regarding the solve time. With a sampling time of 0.02 s, it takes 2.4 times longer on average to solve the NMPC problem, in comparison with the sampling time of 0.05 s. With sampling times of 0.05 s and 0.04 s, the 4WS-TV controller is able to guarantee operation in real time as the solve time is maintained below the boundary. In comparison, the controller cannot run in real time with a sampling time of 0.025 s or 0.02 s due to the solve time exceeding the limit. In summary, the combination of the 0.04 s sampling time and 1.0 s prediction horizon is proven to be able to obtain a good balance between control performance and computational time, and thus is suggested as being suitable for real-time implementation of the proposed 4WS-TV controller.

**Figure 15:**
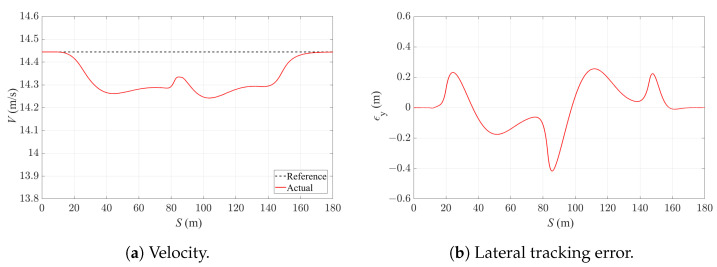
Velocity and lateral tracking error of the vehicle with 4WS-TV controller under real-time implementation with 0.04 s sampling time and 1.0 s prediction horizon.

**Figure 16:**
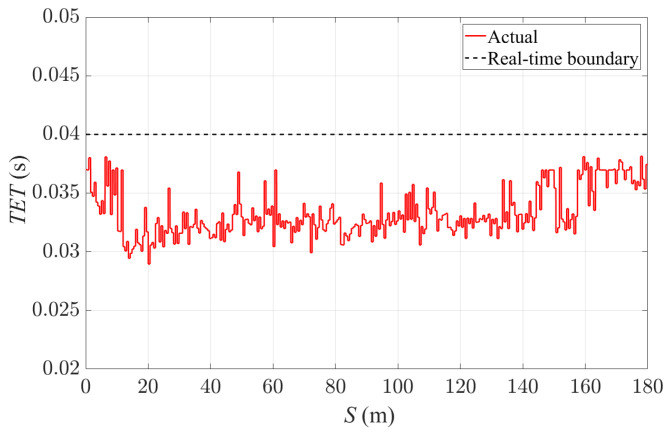
Task execution time of 4WS-TV controller under real-time implementation with 0.04 s sampling time and 1.0 s prediction horizon.

Finally, the 4WS-TV formulation with a 0.04 s sampling time and 1.0 s prediction horizon is implemented on a Speedgoat mobile real-time target machine (Intel Core i7-3555LE CPU at 2.5 GHz with 4 GB RAM and 4MB L2 cache). A 7DoF vehicle model based on [34] with an extension of RWS functionality is used for the real-time simulation. The vehicle dynamics simulation runs at a frequency of 1 kHz. Figure 15a and Figure 15b, respectively, show the velocity-tracking and path-tracking performance of the vehicle in the real-time simulation. The velocity-tracking error is less than 0.2 m/s, while the lateral deviation of the vehicle from the reference path is up to 0.8 m, which is similar to the former simulation results. Figure 16 shows the task execution time (TET) of the control algorithm running in Speedgoat. The capability of the proposed control strategy for real-time execution is validated as the TET remains below the sampling time of the controller. The real-time simulation results prove that the proposed 4WS-TV controller is real-time implementable, and the control performance corresponds with the simulation results in CM.

## 5. Conclusions

In this paper, we have presented a path-tracking controller for multi-actuated autonomous EVs equipped with 4WS as well as TV functionality. The controller design is based on NMPC, and the control formulation has the ability to adapt to different levels of actuation. Four controllers including FWS only, FWS with TV, 4WS and 4WS with TV are constructed and compared in the aspect of path-tracking performance. According to the simulation results, the 4WS-TV controller is able to achieve the best path-tracking performance, especially at the limits of handling condition, by exploiting the multi-actuation capability. When carrying out the same manoeuvre, the maximum lateral deviation of the vehicle from the reference path is around 0.4 m, which is significantly smaller than that with the other three controllers. In addition, with the 4WS-TV controller, there is less oscillation in the vehicle’s yaw rate in transient conditions, which indicates that the application of over-actuation formulation can enhance the vehicle’s stability as well as flexibility at the limits of handling in corners.

Furthermore, it has been proven that the particular level of over-actuation can be implemented in real time with NMPC control formulation, which is another contribution of this paper. In order to reduce system complexity, the impact of prediction horizon and sampling time on the control performance has also been studied. As expected, a longer prediction horizon and shorter sampling time can, in general, have a relatively better path-tracking performance with smaller lateral deviation, at the cost of a higher computational time. Among the different combinations of prediction horizons and sampling times, a compromise has been made between path-tracking performance and computational efficiency, with the combination of a 0.04 s sampling time and 1.0 s prediction horizon time. Finally, the control strategy is implemented in a Speedgoat target machine for real-time simulation, and the simulation results prove that the proposed 4WS-TV controller including the specific nonlinearities of vehicle dynamics is able to run in real time with the particular time parameters while achieving the expected control performance.

In future work, further experimental testing is to be carried out to validate the performance with different levels of actuation. Predictive filtering will also be introduced and investigated as to how that will affect the control performance in response to signal noise and model uncertainty, which is critical in practical implementation. In addition, online path planning will be integrated into the control strategy taking into account the vehicle dynamical capability.

## Figures and Tables

**Table 1:** Actuation topologies of the controllers.

Controller	Steering	Driving Torque
FWS	FWS only	Same on each wheel
4WS	FWS + RWS	Same on each wheel
FWS-TV	FWS only	TV applied on rear wheels
4WS-TV	FWS + RWS	TV applied on rear wheels

**Table 2:** Parameters of the vehicle model.

Parameter	Value	Parameter	Value
*m* [kg]	874.5	*B*	9.50
$I_{z}$ [kg m^2^]	1597.7	*C*	1.63
$l_{F}$ [m]	0.815	*D*	1.16
$l_{R}$ [m]	1.180	$\delta_{F,lim}$ [deg]	19
$w_{L}$ [m]	0.765	$\delta_{R,lim}$ [deg]	19
$w_{R}$ [m]	0.765	$T_{F,lim}$ [Nm]	800
*h* [m]	0.297	$T_{RL,lim}$ [Nm]	350
$R_{w}$ [m]	0.315	$T_{RR,lim}$ [Nm]	350

**Table 3:** Average and maximum lateral deviation of the vehicle with the four controllers.

$t_{s}$ [s]	Average $|\epsilon_{y}|$ [m]	Maximum $|\epsilon_{y}|$ [m]
FWS	0.531	1.337
4WS	0.256	0.684
FWS-TV	0.248	0.642
4WS-TV	0.141	0.404

**Table 4:** Average and maximum lateral deviation of the vehicle with 4WS-TV controller with a fixed sampling time of 0.02 s and different prediction horizon times.

*t* [s]	Average $|\epsilon_{y}|$ [m]	Maximum $|\epsilon_{y}|$ [m]
0.6	0.613	1.747
0.8	0.280	0.783
1.0	0.141	0.404
1.2	0.122	0.454
1.4	0.117	0.508

**Table 5:** Average and maximum lateral deviation of the vehicle with 4WS-TV controller with a fixed prediction horizon of 1.0 s and different sampling times.

$t_{s}$ [s]	Average $|\epsilon_{y}|$ [m]	Maximum $|\epsilon_{y}|$ [m]
0.05	0.164	0.432
0.04	0.151	0.423
0.025	0.145	0.406
0.02	0.141	0.404

## Data Availability

The data supporting this study are included within the article.
